# Graphene Oxide: Preparation and Medical Research

**DOI:** 10.3390/ma18122855

**Published:** 2025-06-17

**Authors:** Xulong Huang, Wengang Zhao, Farid Khalilov, Nuo Xu

**Affiliations:** College of Life and Environmental Science, Wenzhou University, Wenzhou 325035, China; 13780196760@163.com (X.H.); zwg123@139.com (W.Z.); farid.khalilov.87@mail.ru (F.K.)

**Keywords:** graphene, graphene oxide, GO, functionalization, damage, carbon, antibacterial, wound healing

## Abstract

Oxide (GO) has emerged as a highly versatile nanomaterial due to its exceptional physicochemical properties, including large surface area, and strong drug-loading capacity. These characteristics have enabled its broad application in fields such as wound healing, targeted drug delivery, and antimicrobial therapies. However, despite its promise, concerns surrounding GO’s cytotoxicity, biocompatibility, and potential pathological effects have limited its clinical translation. Addressing these limitations requires a deeper understanding of GO’s interactions with biological systems and the development of strategies to mitigate its adverse effects. Recent advances in surface functionalization, covalent crosslinking, and the incorporation of GO into biocompatible matrices have shown great potential in enhancing its performance while minimizing toxicity. This review provides a comprehensive overview of the antibacterial mechanisms of GO and highlights recent progress in chemical modification approaches that improve its efficacy in biomedical applications, particularly in wound healing and drug delivery. By critically examining both the advantages and limitations of GO, this work aims to inform future research directions and support the safe and effective integration of GO-based materials in advanced therapeutic systems.

## 1. Introduction

Throughout one’s life, individuals inevitably encounter both exogenous and endogenous injuries. Improper management of these injuries may cause irreversible harm to the human body; therefore, we encourage injured individuals to promptly choose safe and efficient wound dressings for treatment after injury.

Graphene has been a trend in physics and electronics since its discovery in 2004 [1]. Graphene oxide (GO), as an oxidized derivative of graphene, has attracted more attention as a variety more suitable for chemical modification and biological functionalization with the rise of the graphene material boom. Due to its unique two-dimensional nanostructure, high surface area ratio, good biocompatibility, and adjustable surface chemical properties, GO has become a research hotspot in the field of biology/medicine since 2012.

In the medical domain, GO’s multifunctionality demonstrates remarkable potential for mitigating exogenous damage through multiple mechanisms: (1) the suppression of inflammatory cascades and immune microenvironment modulation [1,2], (2) improving the microenvironment of wounds [1,3], (3) the development of stimuli-responsive drug delivery systems [4], (4) broad-spectrum antimicrobial/antiviral actions [3], and (5) ultrasensitive biosensing for disease biomarkers [5,6]. Owing to its versatile physicochemical properties, graphene oxide (GO) has attracted significant attention as a multifunctional platform. Researchers have been motivated to integrate GO with a wide range of materials to fully harness its potential. Beneficially, GO possesses abundant oxygen-containing functional groups (e.g., hydroxyl, epoxide, carboxyl) that enable a variety of chemical modifications and surface functionalization strategies. These features facilitate its effective combination with polymers, biomolecules, nanoparticles, and other functional agents. As a result, GO has emerged as one of the most actively investigated two-dimensional materials in fields such as wound healing, drug delivery, antimicrobial applications, and microscale sensing.

## 2. Structure and Mechanism of Graphene Oxide

### 2.1. Physical Properties of GO

Graphene-based material showcases distinctive physical, chemical, and biological properties, encompassing unique optical and exceptional transparency. Graphene-based material has good strength and is remarkably lightweight [7]. It features an extensive surface area reaching up to 2630 square meters per gram [8,9], providing an extraordinary capacity for drug loading. At room temperature, graphene-based material exhibits exceptional thermal conductivity [10].

**Figure 1:**
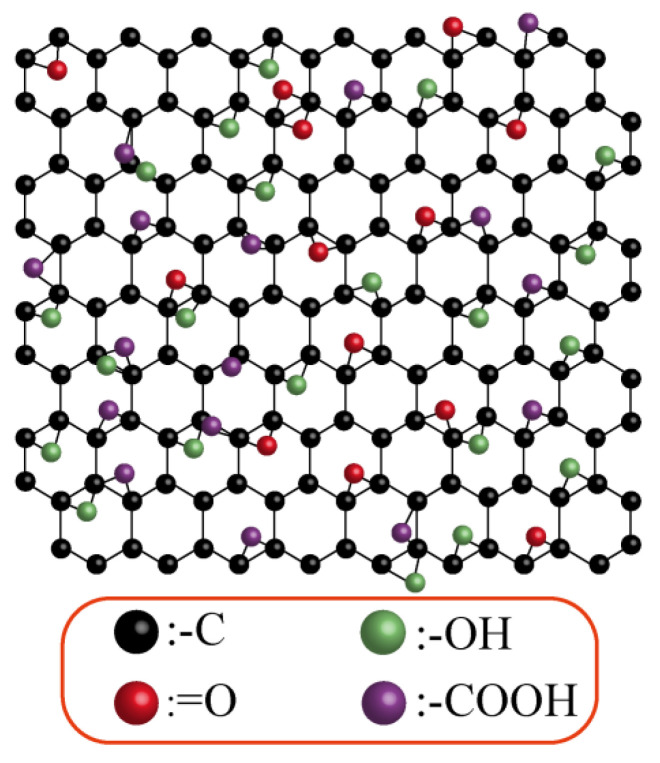
GO structure diagram.

Graphene oxide (GO), an extensively oxidized derivative of graphene (Figure 1), typically exhibits a carbon-to-oxygen atomic ratio of 1.5–2.5 while preserving its characteristic honeycomb lattice structure [11,12]. Owing to its remarkable multifunctionality and ability to modulate cellular behavior, GO has emerged as an ideal scaffold material in regenerative medicine. GO-based constructs not only withstand physiological mechanical loads but also enhance cell adhesion, proliferation, and lineage-specific differentiation. In particular, composite hydrogels formed from GO and natural polymers combine superior mechanical strength with intrinsic bioactivity, demonstrating promising outcomes in soft tissue repair. Moreover, the large specific surface area of GO facilitates high drug-loading capacity and protects therapeutic agents from degradation and inactivation under physiological conditions, thereby substantially prolonging drug stability and release duration in delivery systems.

Graphene oxide (GO) exhibits exceptional sensitivity and ultralow detection limits, underscoring its transformative potential in the field of biosensing. The abundance of oxygen-containing functional groups on its surface not only facilitates the immobilization of recognition elements but also enhances sensing specificity and accelerates response kinetics. These properties make GO a promising candidate for real-time and highly selective biomarker detection. In the context of oncotherapy, GO also demonstrates remarkable photothermal conversion efficiency, positioning it as a valuable agent for photothermal therapy and multifunctional theragnostic applications. Under near-infrared light irradiation, GO can efficiently convert light energy into heat, which increases the local temperature and kills cancer cells. Studies have shown that GO exhibits excellent performance in photothermal therapy [13]. Ding et al. developed a conductive graphene oxide (GO)-based hybrid hydrogel scaffold that exhibits photothermal responsiveness under near-infrared (NIR) light. Upon NIR irradiation, the elevated temperature induces the cleavage of thermosensitive C=C double bonds within the GO structure, resulting in a marked reduction in the scaffold’s mechanical stiffness. Simultaneously, this structural change facilitates the controlled release of drugs embedded within the hydrogel matrix. Additionally, the heat generated during NIR exposure imparts the scaffold with notable antibacterial properties. This NIR-responsive hydrogel introduces a novel strategy in the biomedical field by enabling drug release through thermally triggered structural transformation, while concurrently achieving localized antibacterial effects [4].

The abundance of hydroxyl and carboxylic acid functional groups endows graphene oxide (GO) with pronounced hydrophilicity [14,15], thereby enhancing its biocompatibility. These functional groups also facilitate interactions and binding with a wide range of molecules and polymers, making GO highly attractive for drug delivery applications. Following surface functionalization—leveraging its large surface area and π-conjugated structure [16]—GO can adsorb various proteins without compromising their structural integrity while maintaining excellent biocompatibility in cell adhesion. GO interacts with proteins via both covalent and non-covalent mechanisms. Covalent interactions typically involve chemical reactions between amino acid side chains and the functional groups on GO’s surface, while non-covalent interactions may occur through hydrophobic forces or shape-complementary interactions with hydrophobic domains of proteins [17]. These versatile interaction modes contribute to GO’s growing popularity in biomedical applications, particularly in drug delivery and tissue engineering [18,19,20].

Owing to its high surface area and rich surface functionalization, graphene oxide (GO) offers an effective platform for drug delivery by enabling high drug-loading capacity and strong adhesion to cellular membranes through various physicochemical interactions, thereby facilitating controlled and targeted drug release. Furthermore, when combined with other materials, GO can be incorporated into intelligent hydrogel systems that respond to external stimuli such as electrical signals or photothermal changes. These smart hydrogels are capable of rapid and precise responses to pathological stimuli—such as infection risks or sudden surges in exudate—enabling real-time therapeutic intervention. Collectively, these properties position GO as a highly promising material for applications in connective tissue regeneration [7].

Recent research has highlighted graphene oxide (GO) as a promising drug delivery platform with distinct advantages in tissue regeneration. Its ability to form a three-dimensional network structure enables controlled and sustained drug release, while simultaneously modulating the local microenvironment to promote cellular proliferation and differentiation. This dual functionality provides multidimensional support for dynamic tissue repair, positioning GO as a versatile material in regenerative medicine. A representative case is the bioinspired composite hydrogel system developed by Eunkyoung Byun’s team. Utilizing mussel-inspired catecholamine chemistry, researchers integrated GO nanosheets into a hyaluronic acid (HA) hydrogel network. This HA–GO hybrid system achieves efficient drug loading (doxorubicin loading efficiency > 89%) through passive diffusion mechanisms and maintains stable sustained-release kinetics (cumulative release rate 90%) over 10 days, significantly enhancing therapeutic outcomes in damaged tissues [21]. Liang et al. developed a smart pH- and glucose-responsive hydrogel capable of dynamically releasing metformin in response to the fluctuating physiological conditions characteristic of different stages of diabetic wound healing. This targeted and stimuli-responsive release profile significantly enhanced the wound repair process. Beyond its controlled drug delivery function, the hydrogel demonstrated a range of beneficial properties, including rapid hemostasis, potent antibacterial and antioxidant activities, and strong tissue adhesion. These multifunctional attributes highlight the hydrogel’s considerable potential for clinical application in the treatment of chronic wounds, particularly in the context of diabetic wound management [22].

### 2.2. Antibacterial Ability

GO exhibits notable antibacterial properties [21], primarily through mechanisms involving membrane disruption, oxidative stress induction, and the physical entrapment of bacterial cells [23]. The disruption of bacterial membranes by GO is multifactorial. One of the primary mechanisms is the so-called “nano-blade” effect, in which the sharp edges of GO’s two-dimensional structure physically pierce and compromise the integrity of bacterial membranes, leading to the leakage of intracellular content and subsequent cell death [24]. In addition to mechanical disruption, the presence of oxygen-containing groups on the GO structure triggers oxidative stress when they interact with bacterial cells. This interaction prompts the generation of reactive oxygen species (ROS) within the cells. These ROS molecules subsequently overwhelm the antioxidant capacity of the bacterial cells, exacerbating cellular damage and contributing to cell inactivation [25]. It is worth noting that this phenomenon is often initiated by the chemical reduction of graphene sheets facilitated by bacterial enzymes or metabolic processes. Furthermore, GO exhibits the ability to physically capture and encapsulate bacterial cells, effectively trapping them within its structure. This process disrupts the connection between the bacterial cell and its external environment, leading to alterations in cell metabolism, inhibition of cell proliferation, and induction of cell apoptosis [26]. This mechanism is particularly prominent when GO is dispersed in a solution where it can actively interact with suspended bacterial cells. Interestingly, wrinkled GO sheets have been observed to possess an enhanced capability in capturing bacterial cells. This enhanced interaction between wrinkled GO and bacteria leads to heightened membrane stress, membrane disruption, leakage of cellular components, and, ultimately, cell lysis [24]. Collectively, these findings highlight the multifaceted and potent antibacterial mechanisms of GO, underscoring its significant potential in antimicrobial applications.

### 2.3. Cytotoxicity

It is important to note that although graphene oxide (GO) exhibits antibacterial activity, it also inevitably poses cytotoxic risks to mammalian cells. However, reliable studies have shown that when the concentration of graphene oxide solution is less than 20 μg/mL, it is non-toxic to human fibroblasts. When the concentration exceeds 50 μg/mL, the solution exhibits significant toxicity, including reduced cell survival rate, decreased cell adhesion, and significant changes in cell morphology. This level also occurs in mice. Graphene oxide solution has no significant toxicity to mice at 0.25 mg, but when it exceeds 0.4 mg, GO solution shows toxic effects, leading to accumulation in the lungs, liver, spleen, and kidneys of mice and subsequently their death. The results indicate that the toxicity of graphene oxide is dose-dependent. When the concentration of graphene oxide is below a certain value, there will be no cytotoxicity [27,28,29,30,31]. Additionally, the surface modification of GO has emerged as an effective strategy to further enhance its biocompatibility and mitigate its adverse effects [32,33,34].

Covalent crosslinking of graphene oxide (GO) with biocompatible polymers such as chitosan, poly(acrylic acid) (PAA), or polyethylene glycol (PEG) significantly reduces its cytotoxicity while simultaneously promoting cell adhesion and proliferation. This improvement is primarily attributed to the effective masking of GO’s sharp edges by the polymer matrix, which minimizes direct mechanical damage to cell membranes. Additionally, the increased particle size of the resulting GO–polymer composites may impede the cellular internalization of GO, thereby further mitigating its potential cytotoxic effects [27,35,36,37].

Recent studies have demonstrated that graphene oxide (GO) can promote angiogenesis, a critical process in tissue regeneration and wound healing. This pro-angiogenic effect is believed to be mediated through the intracellular generation of reactive oxygen species (ROS) and reactive nitrogen species (RNS), which in turn activate endothelial nitric oxide synthase (eNOS) and the phosphorylation of protein kinase B (Akt). Collectively, these processes stimulate the nitric oxide (NO) signaling pathway, which plays a vital role in vascular development. Depending on the concentration of GO or reduced graphene oxide (rGO) and the corresponding levels of ROS production, these nanomaterials may either enhance or inhibit angiogenesis. When appropriately dosed, GO has been shown to effectively promote neovascularization, thereby contributing to accelerated skin wound healing by enhancing blood vessel formation [38,39,40].

### 2.4. Structure

Various models of graphene oxide (GO) structures have been proposed, and these models encompass both two-dimensional (2D) and three-dimensional (3D) configurations. They include the Hofmann, Ruess, Scholz–Boehm, Nakajima–Matsuo, Lerf–Klinowski, and Szabo models. Among these, the Lerf–Klinowski model proposed by Lerf and colleagues stands as the most widely accepted model. Lerf and colleagues utilized ^13^C and ^1^HNMR spectroscopy to validate the presence of hydroxyl groups, epoxy groups, and carbon–carbon double bonds in GO, properties that form the foundation of their proposed model.

According to the Lerf–Klinowski model, graphene oxide (GO) consists of two structurally and chemically distinct regions [12]: The first is the non-oxidized aromatic domain, composed primarily of sp^2^-hybridized carbon atoms, which retain the conjugated structure characteristic of pristine graphene. The second is the oxidized aliphatic domain, composed of sp^3^-hybridized carbon atoms within six-membered ring structures bearing oxygen-containing functional groups such as hydroxyl and epoxide. The relative proportions and sizes of these regions depend on the degree of oxidation. Importantly, the oxidation sites are randomly distributed across the surface of GO, making precise control of their location challenging. Due to this heterogeneous functionalization, the edges of GO sheets tend to be hydrophilic—rich in carboxyl and hydroxyl groups—while the basal planes remain relatively hydrophobic. This dual character imparts amphiphilic properties to GO, enabling it to behave like a large, sheet-like surfactant molecule in various environments.

Apart from the hydroxyl groups, which induce slight twisting and wrinkling of the plane, graphene oxide (GO) largely maintains a flat two-dimensional (2D) structure. It is characterized by the presence of putative1,3-epoxy and hydroxyl groups dispersed across the layers of GO and at the edges because of their low concentrations. Advancements in detection techniques, such as Fourier transform infrared (FT-IR), X-ray photoelectron spectroscopy (XPS) [41], X-ray powder diffraction (XRD) [8], and near-edge X-ray absorption fine structure (NEXAFS) spectroscopy [42,43,44], have verified the existence of epoxy groups, hydroxyl groups, ketone groups, sulfate esters, and other functional groups on GO. Observations of GO layers via scanning electron microscopy (SEM) have revealed highly disordered oxidized regions alongside non-oxidized graphite regions. Additionally, defects such as excessive oxidation and pore formation during layer delamination have been identified [45].

Graphene oxide (GO) nanoparticles inherently exhibit significant heterogeneity, manifested in variations in particle number, lateral dimensions, surface chemistry, defect density, sheet quality, and overall composition or purity. Such inconsistencies present substantial challenges in both reproducibility and performance, particularly for biomedical applications such as targeted drug delivery. These variations can critically influence drug loading efficiency, release kinetics, and biocompatibility. Therefore, the development of a reliable and scalable synthesis strategy that enables precise control over the size, structural integrity, and surface properties of GO nanoparticles is urgently needed. Such advancements would pave the way for the standardized production of GO with optimized characteristics tailored to specific therapeutic applications [46]. McAllister et al. demonstrated that the lateral size of graphene oxide (GO), produced from fully oxidized graphite, is independent of the initial size of the graphite particles, suggesting that graphite particle size is not the primary determinant of GO sheet dimensions. In contrast, Zhao et al. reported that the degree of oxidation, controlled via the Hummers method, plays a crucial role in governing the size of GO sheets. By modulating oxidation conditions and applying ultrasonication, they successfully synthesized GO with a significantly increased surface area. Furthermore, Li et al. observed that the formation of epoxy groups during oxidation can weaken interlayer van der Waals interactions, facilitating exfoliation. They proposed that an increased density of carbon–oxygen bonds leads to a reduction in sheet size due to the development of cracks on hydroxyl- and epoxy-functionalized surfaces. Collectively, these studies indicate that the lateral dimensions of GO can be tuned not only by balancing the oxidative penetration from the sheet edges with the kinetics of crack propagation but also by precisely controlling the extent of oxidation [47].

## 3. Preparation and Modification of Graphene Oxide

Graphene oxide (GO) has emerged as a key material in interdisciplinary research due to its distinctive two-dimensional layered structure, tunable surface functional groups, and exceptional physicochemical properties. To achieve efficient GO production, various oxidation–exfoliation strategies have been developed over time, with the Brodie (1859) [48], Staudenmaier (1898), and Hummers (1958) [49] methods being the most representative classical approaches. Among them, the Hummers method has gained the most widespread adoption due to its relatively high efficiency, shorter reaction time, and safer operational conditions. Therefore, the following discussion will focus primarily on the Hummers method.

### 3.1. The Hummers Method

Compared to the Brodie and Staudenmaier methods, the later-developed Hummers method [4] is simpler, more efficient, and offers significant improvements in product quality and yield [4]. Due to its enhanced safety profile and scalability, the Hummers method has become the most widely adopted approach for graphene oxide synthesis. Originally proposed by American chemist Richard E. Hummers in 1958, this method employs strong oxidants—typically sulfuric acid, nitric acid, and potassium permanganate—to oxidize graphite while introducing oxygen-containing functional groups in a relatively controlled and safer manner. The resulting graphene oxide exhibits stable quality, high purity, and good reproducibility, making it suitable for producing high-quality GO materials. The following section provides an overview of the Hummers method for graphene oxide preparation.

**Figure 2:**
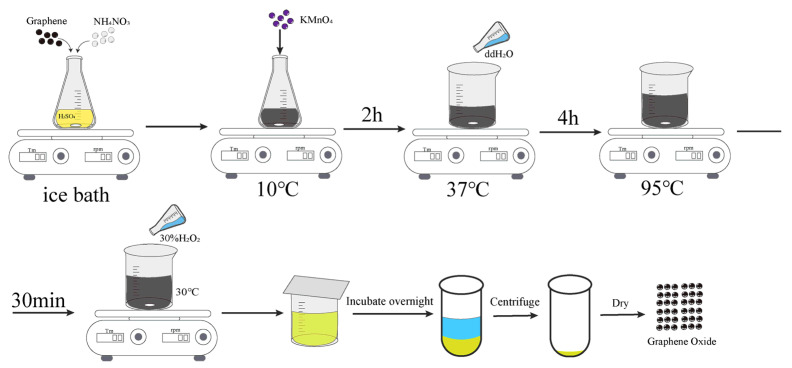
Preparation diagram of GO.

Firstly, concentrated sulfuric acid is added to a conical flask placed in an ice bath. Graphite powder and sodium nitrate are then introduced, and the mixture is stirred while maintaining a low temperature for 2 h. During this period, a controlled amount of potassium permanganate is slowly added to the reaction. After 2 h, the mixture is transferred to a 500 mL beaker and placed in a water bath maintained at 35–38 °C with continuous stirring. Subsequently, 100 mL of distilled water is added slowly, and the solution is diluted to a total volume of 300 mL and maintained at medium temperature for 4 h. Following this, the reaction mixture is heated to 90–95 °C and stirred for an additional 30 min. After completion, the beaker is removed and allowed to cool to room temperature (approximately 30 °C). Then, 30% hydrogen peroxide solution is added until the color changes to bright yellow, indicating the reduction of residual permanganate ions. The mixture is sealed and allowed to stand overnight. The next day, the solution is centrifuged, and the precipitate is washed with 5% dilute hydrochloric acid to remove sulfate ions, followed by repeated washing with distilled water until a neutral pH is reached. The resulting product is exfoliated using ultrasonication for 1 h, followed by low-speed centrifugation to separate the graphene oxide (GO) sheets. Finally, the material is dried to obtain the GO sample (Figure 2).

The proposal of the Hummers method marks a significant advancement in the synthesis of graphene oxide. The widespread application of the Hummers method has also greatly promoted the research and application of graphene oxide in various fields [17,50].

In a word, the Hummers method is the most classic and widely used preparation method of graphene oxide at present. By introducing the mixed system of potassium permanganate and concentrated sulfuric acid, it can efficiently oxidize graphite in a short time and avoid the risk of a strong reaction caused by the use of fuming nitric acid and potassium chlorate in the early method, which significantly improves the safety and convenience of the experiment. This method not only provides a stable way for the large-scale and controllable synthesis of graphene oxide but also lays an important foundation for the further application of GO in many fields such as structural regulation, surface functionalization, and biomedicine [51,52,53].

### 3.2. Graphene Oxide Modification

The proposal of the Hummers method provides an efficient, stable, and safe technical foundation for the large-scale preparation of graphene oxide (GO), making it the most commonly used precursor material in research and application. To further expand the application potential of GO in composite systems, researchers functionalized it by introducing specific functional groups such as amino, carboxyl, thiol, etc. [31,32,33]. This type of modification strategy not only significantly enhances the interfacial binding ability of GO with biomolecules, metal ions, or polymer materials but also endows it with more precise functional characteristics in fields such as biometric recognition, sensing and detection, catalysis, and drug delivery [27,34,35], fully tapping into the unique advantages of GO as a multifunctional nanoplatform.

The functionalization of the abovementioned oxygen-containing is achieved by chemical modification via the formation of covalent bonds, resulting in functionalized GO. This process can be categorized into two types: edge functionalization involving carboxyl groups and basal plane functionalization involving hydroxyl and epoxy groups. Typically, GO functionalization is employed in the construction of GO-based polymer composite materials with the aim of augmenting their thermal and mechanical stability.

The abovementioned oxygen-containing can be prepared by either the covalent modification of functional groups or by non-covalent interactions, which rely on hydrogen bonds and van der Waals forces between polymers and GO. The main pathway for the covalent modification of dispersed liquids is grafting, which involves connecting one or more chemical groups to a chemical modification layer on the material surface, covalently linking the chemical groups to the material surface to form a new grafting layer, thereby endowing the material surface with new characteristics and properties. The active sites located at the boundaries or defect sites on GO can be first identified and then covalently linked with the desired substance to graft it onto GO to achieve the desired materials. Typically, the molecular weight of the modified material is used to distinguish between modifications involving polymers and organic small molecules. Functionalized graphene and GO not only have enhanced dispersibility in solvents but also improved amphiphilicity and mechanical properties. Furthermore, the prepared modified GO finds applications in the field of biology, thereby expanding the potential application range of graphene.

Rabchinskii et al., by improving the Leucart reduction amination reaction, converted graphene oxide (GO) into aminated graphene (rGO Am), and a chemical resistance biosensor composed of a graphene oxide aerosol deposition layer and conjugated antibody was prepared and tested. In the detection of IgM immunoglobulin, the detection limit was as low as 10 pg/mL. This excellent performance can make graphene oxide occupy a strong position in high-sensitivity detection [5].

## 4. Graphene Oxide Composites

### 4.1. Graphene Oxide Nanoparticles

At present, the common types of nanomaterials mainly include metal-based nanomaterials [54], polymer nanoparticles [14,55,56], carbon-based nanomaterials [57], and nano gel [58]. Among them, carbon-based nanomaterials have become a research hotspot in the biomedical field due to their unique physical and chemical properties and multifunctionality. Graphene oxide (GO), as a typical carbon-based two-dimensional nanomaterial, is formed by the oxidation and exfoliation of graphite flakes. It not only has a high specific surface area and controllable surface functional groups but also has broad application prospects in the field of biomedicine due to its good biocompatibility and ease of functional modification.

Notably, graphene oxide, as a type of carbon-based nanomaterial, stands out as a two-dimensional material derived from the exfoliation of graphite flakes, featuring surface functional groups such as hydroxyl (-OH), carboxyl (-COOH), and aldehyde (-CHO). These oxygen-containing functional groups confer upon GO distinctive properties, including hydrophilicity and antimicrobial activity, enabling GO to engage in facile reactions with both organic and inorganic materials. Therefore, various nanocomposites with unique properties can be synthesized using the square characteristics of GO, which have been widely used in drug delivery, photothermal diagnosis and treatment, wound repair, and other fields [59,60,61].

### 4.2. Graphene Oxide Hydrogel

When GO is used as the preparation material of a hydrogel, graphene oxide can give full play to its advantages. For example, it can improve the drug molecules that hydrogel can carry through its rich functional groups and large surface, improve the strength of hydrogel through its physical properties, endow hydrogel with the possibility of responding to environmental stimuli, and endow hydrogel with antibacterial ability through its excellent antibacterial effect.

When graphene oxide (GO) is incorporated as a component in hydrogel fabrication, it can fully leverage its unique advantages. For instance, GO’s abundant functional groups and large specific surface area enhance the hydrogel’s drug-loading capacity. Its exceptional physical properties contribute to the improved mechanical strength of the hydrogel. Additionally, GO can impart environmental responsiveness to the hydrogel, enabling stimuli-sensitive behavior. Furthermore, the intrinsic antibacterial activity of GO endows the hydrogel with effective antimicrobial properties [62].

Song et al., by modifying graphene oxide to nitro–graphene oxide derivatives, increased the oxidation level, antibacterial activity, and dispersion. Through repeated freezing and thawing, hydrogels with high antibacterial activity that promoted wound healing were prepared. Compared with GO hydrogel, the antibacterial effect of the modified NGO hydrogel against Escherichia coli and Staphylococcus aureus was increased by about four times, and the bactericidal rates were 99.72% and 99.38%, respectively [63].

### 4.3. Graphene Oxide Composite Material

In fact, graphene oxide also has excellent performance in the field of chemistry. Graphene oxide (GO) features abundant oxygen-containing functional groups on its surface, which can be converted to reduced graphene oxide (rGO) via a disproportionation reaction. The presence of epoxy functional groups endows GO with good dispersibility and stability in water or other solvents. Moreover, numerous oxygen-containing functional groups at the edges of GO render it highly hydrophilic, while the plane is relatively hydrophobic, imparting amphiphilic properties. However, in non-polar solvents like xylene, the dispersibility of graphene oxide diminishes significantly because of strong π–π interactions between layers and the presence of robust van der Waals forces, resulting in severe layer aggregation. This aggregation impedes the excellent performance of graphene as a material. Consequently, the surface modification of graphene oxide is imperative to address these challenges.

Organic high-molecular-weight polymers, such as polyaniline, polyvinylpyrrolidone, and polyethylene, have been extensively employed to modify graphene oxide (GO) due to their ability to improve dispersibility through steric stabilization. The incorporation of these polymers increases the interlayer spacing of GO sheets, thereby introducing steric hindrance that effectively suppresses sheet restacking and aggregation. Among various chemical strategies, esterification reactions are widely utilized for the macromolecular modification of GO. For instance, Cheng et al. reported the successful grafting of polyvinyl alcohol (PVA) onto GO nanosheets via esterification, establishing a novel approach for the fabrication of high-performance polymer nanocomposites by incorporating PVA-grafted GO into a PVA matrix [64]. The resulting composites exhibited markedly improved mechanical properties, which were attributed to the strong interfacial adhesion and compatibility between PVA–g-GO and the PVA matrix. This enhancement was primarily driven by extensive hydrogen bonding interactions and structural compatibility between the two components [64].

The modification of the functional groups of graphene and GO serves a dual purpose. It enhances the dispersibility of the modified graphene or GO in solvents while improving their amphiphilicity and mechanical properties. Additionally, the modified GO also finds utility in the realm of biology, expanding the potential application scope of graphene. However, in the modification of organic macromolecules, the interaction between the functional groups of the molecule and the surface of GO disrupts the continuity of the original GO structure, resulting in the suppression of its conductivity and optical properties.

## 5. Applications of Graphene Oxide in the Medical Field

### 5.1. Application of Graphene Oxide in Antibacterial Field

The use of antimicrobial agents, particularly antibiotics, has played a crucial role in human history in controlling microbial infections. However, the pipeline for developing novel antimicrobial compounds has slowed significantly in recent decades. This stagnation, coupled with the global overuse and misuse of antibiotics, has driven the emergence of sophisticated resistance mechanisms in pathogenic microbes. These adaptive strategies enable microorganisms to survive under antibiotic pressure, leading to an alarming acceleration of antimicrobial resistance worldwide. The rise of resistant pathogens presents grave challenges for both public health and industrial applications. Bacterial populations employ multiple strategies to acquire resistance, including horizontal gene transfer through which they exchange genetic material. For instance, Escherichia coli, a common foodborne pathogen, demonstrates remarkable genomic plasticity with approximately 80% of its DNA capable of recombination and foreign gene incorporation. While some bacterial species possess intrinsic multidrug resistance, others develop it through spontaneous mutations that are then propagated to daughter cells during replication. Conventional antimicrobial agents are increasingly ineffective against these dynamic resistance mechanisms. Their inability to counteract bacterial adaptation strategies has created an urgent need for innovative approaches that can overcome microbial evolutionary defenses. New therapeutic paradigms must address not just bacterial viability but also their capacity for rapid genetic adaptation.

In 2010, Huang et al. first reported the antibacterial properties of graphene oxide (GO) [65]. Subsequent studies have identified several primary antibacterial mechanisms attributed to GO, including the induction of oxidative stress, physical and mechanical damage to bacterial membranes, photothermal and photocatalytic effects, inhibition of bacterial metabolism, lipid extraction, and bacterial isolation through encapsulation. Both large and small-sized GO sheets contribute to antibacterial efficacy, albeit via different mechanisms: the encapsulation and lipid extraction effects are more prominent with larger GO sheets, while the physical cutting and oxidative stress mechanisms are primarily associated with smaller GO sheets [66].

In recent years, graphene oxide (GO)-based nanocomposites have attracted considerable attention across diverse application domains. Functionalization strategies have proven effective in enhancing the antibacterial properties of GO [67,68]. Notably, Yu et al. pioneered the synthesis of a GO–polyethyleneimine (PEI)–silver nanoparticle (AgNP) hybrid using a microwave-assisted method. They subsequently incorporated this composite into polyacrylonitrile (PAN) nanofibers via electrospinning, resulting in core–shell structured nanofibers. The fabricated fabric exhibited outstanding antibacterial efficacy, achieving inhibition rates exceeding 99.99% against both Escherichia coli and Staphylococcus aureus. Remarkably, the antibacterial performance remained above 99.99% even after 10 washing cycles, highlighting both high antimicrobial activity and long-term durability. A growing body of research further supports the role of GO as a synergistic antibacterial agent, leveraging its large specific surface area and distinctive physicochemical characteristics to enhance the antimicrobial efficacy of composite materials [69].

### 5.2. Application of Graphene Oxide in Skin Wounds

Graphene oxide exhibits exceptional antibacterial properties that show great promise for skin wound healing applications. As the body’s primary barrier against external pathogens, the skin requires effective protective measures during wound recovery. Graphene oxide’s inherent antimicrobial characteristics make it particularly suitable for wound treatment applications. Beyond its antibacterial effects, graphene oxide’s large surface area and unique three-dimensional structure significantly enhance drug transdermal permeability. This property facilitates more efficient drug penetration through the skin barrier into systemic circulation, substantially improving drug delivery efficacy. When utilized as a drug carrier, graphene oxide enables precise control over drug release kinetics through structural modulation. This controlled-release capability reduces the need for frequent dressing changes, thereby minimizing wound disturbance and promoting optimal healing conditions. These advantageous properties have led to growing applications of graphene oxide in advanced wound dressings, where it demonstrates dual functionality in both infection control and tissue regeneration support. Its multifunctional nature positions graphene oxide as a highly promising material for next-generation wound care solutions [70]. Wound dressings, including electrospun mats [71], hydrogels [72], and sponges [73], are among the best options for accelerating wound healing.

Currently, the surface modification of graphene oxide (GO) and its integration with functional compounds or polymeric materials have emerged as a prevalent and effective strategy for developing GO-based wound dressings. Through the chemical modification of surface functional groups (e.g., carboxyl, hydroxyl, and epoxy groups), GO can achieve improved material compatibility and stability while gaining enhanced bioactivity or stimulus-responsive functionality. Compared to their original substrates, these GO–composite dressings demonstrate superior overall performance, particularly in mechanical strength, antibacterial efficacy, and wound healing promotion, making them promising candidates for advanced wound care applications.

Specifically, the addition of GO can significantly enhance the mechanical properties of dressings, such as tensile strength and ductility, making them more flexible and durable during skin or wound application [74]. Meanwhile, GO’s ability to regulate oxidative stress and its destructive effect on bacterial cell membranes endow the material with excellent broad-spectrum antibacterial properties, effectively inhibiting the growth of common pathogenic bacteria such as Escherichia coli and Staphylococcus aureus. In addition, multiple studies have shown that GO can regulate cell behavior and promote fibroblast proliferation and collagen production, thereby accelerating wound closure and the healing process [38,75,76].

### 5.3. Application of Graphene Oxide in Drug Delivery

Graphene oxide, due to its unique two-dimensional structure, high specific surface area, and abundant surface functional groups, has great potential as a nanocarrier in the field of drug delivery. Specifically, GO’s π–π stacking can bind to drug molecules with aromatic rings. This stacking is a medium-strength non-covalent bond. When the drug is in an acidic environment, the protonation of the drug molecule will cause a change in charge, thereby weakening the π–π stacking and promoting targeted drug release. The carboxyl group (-COOH) of GO can form amide bonds with the amino group (-NH_2_) of proteins through crosslinking agents such as EDC/NHS, achieving covalent fixation (such as antibody and enzyme fixation). Hydroxyl (-OH) can form hydrogen bonds with polar groups of drug molecules, such as hydroxyl and amino groups. Epoxy (O-) can react with amino or thiol groups under alkaline conditions to form covalent bonds. Epoxy groups can also serve as anchoring points for grafting functional molecules (such as polyethylene glycol PEG) through chemical reactions. GO can also introduce functional groups (-NH_2_-SH, -SO_3_H) and react with other drugs to form covalent bonds. The single atomic layer two-dimensional structure of GO exposes all its atoms on the surface, providing uniform and extensive binding sites for drug molecules. When multiple layers of GO are stacked, nanoscale gaps can be formed between the layers, and drugs can be loaded through physical adsorption or chemical bonding. As mentioned above, the specific surface area of GO can reach up to 2600 m^2^/g, far exceeding traditional carriers (such as liposomes~100 m^2^/g, mesoporous silica~1000 m^2^/g). Such an exaggerated surface area means that GO can adsorb more drug molecules compared to other materials. The following table shows the applications and effects of graphene oxide as a delivery material in recent years (Table 1).

### 5.4. The Popularity of Graphene Oxide in Wearable Devices

Graphene oxide (GO) has garnered increasing attention in advanced material research due to its unique combination of tunable electrical conductivity, mechanical flexibility, large specific surface area, and excellent biocompatibility. Unlike pristine graphene, GO contains abundant oxygen-containing functional groups, which not only allow for enhanced dispersion in aqueous systems and facile chemical modification but also enable integration with various polymers and substrates. These properties make GO particularly well-suited for applications requiring both electrical functionality and structural adaptability. In the context of smart wearable electronics, GO’s flexibility and processability facilitate its use in the fabrication of lightweight, stretchable, and conformable components such as biosensors, conductive textiles, and flexible circuits. Furthermore, its ability to form stable interfaces with biological tissues supports its use in continuous physiological monitoring and human–machine interfacing. Collectively, these attributes position GO as a highly versatile material platform for the next generation of intelligent, wearable technologies.

Gao et al. successfully designed and manufactured a skin-based flexible gel electrolyte graphene transistor (GEGT) for glucose detection. Their sensor uses glycerin gel instead of traditional liquid electrolyte, which can not only better fit human skin but also play a role in liquid collection. It has good selectivity and anti-interference ability for glucose and can detect at least 10 nM glucose. Moreover, the GEGT sensor has good stability, which can stably monitor electrophysiological signals for a long time, even in dry environments, and has broad application prospects in the field of non-invasive wear for smart medical monitoring [83].

### 5.5. Application of Graphene Oxide in Bone Regeneration

In the realm of bone regeneration, GO plays a pivotal role by facilitating essential alkaline phosphatase activity, calcium deposition, and enhancing biocompatibility. This has garnered significant attention for its applications in scaffolds, coatings, tympanic membrane guidance, and drug delivery systems. One notable advantage of graphene oxide materials in skeletal applications is their capacity to induce osteogenic differentiation, thereby fostering accelerated bone repair. Additionally, graphene oxide exhibits antibacterial activity while preserving the viability, attachment, and proliferation of osteoblasts [7].

Ink made of gelatin methacrylate, polyethylene glycol diacrylate, and graphene oxide represents a novel cartilage printing material. When induced by graphene oxide, hMSCs (human mesenchymal stem cells) exhibit enhanced chondrogenic differentiation, leading to increased levels of glycosaminoglycan and collagen. Seifalian and colleagues dedicated their research to the construction of ear cartilage for children with microtia, a congenital deformity of the ear [84].

Yeqiao et al. developed PVA–GO-PEG nanocomposite hydrogels using a freeze–thaw method. The presence of polyethylene glycol (PEG) led to the efficient grafting of polyvinyl alcohol (PVA) molecules onto the surface of graphene oxide (GO). The tensile strength, elongation at break, and compressive modulus were all improved. Additionally, for samples with 1.5% graphene oxide content, both maximum force retention and dynamic stiffness are increased. In the composite hydrogels, the friction coefficient decreases by over 50%. A small amount of nanographene oxide (NGO) can enhance the mechanical and biomedical properties of microionically cross-linked gelatin-based hydrogels, facilitating transparent cartilage formation without the need for expensive growth factors [85].

## 6. Conclusions

Graphene oxide (GO) demonstrates significant potential for precision drug delivery applications owing to its abundant surface functional groups and exceptionally high specific surface area. These structural characteristics enable effective drug loading and targeted release. Additionally, GO possesses remarkable thermal conductivity along with unique physicochemical and biological properties, making it particularly suitable for developing highly sensitive chemical and biological sensors. The material’s exceptional antibacterial properties further contribute to its biomedical value, showing significant efficacy in infection prevention and biofilm inhibition. These multifunctional advantages position GO as a highly promising material for advancing biomedical technologies. While GO exhibits dose-dependent toxicity, proper concentration control can ensure its safe application in medical treatments. Notably, substantial research evidence confirms GO’s ability to promote angiogenesis, adding to its therapeutic potential. Consequently, GO continues to attract considerable research attention in biomedicine. Future investigations are expected to yield a deeper mechanistic understanding of GO, leading to more extensive development and broader clinical applications. This progress will undoubtedly enhance our knowledge of GO’s biological interactions and accelerate its translation into practical biomedical solutions.

## Figures and Tables

**Table 1:** Delivery of graphene oxide.

Delivery Materials	Delivery of Drugs	Effect	Ref.
CS@GO/Fe_3_O_4_	camptothecin (CPT)	The highest release rate reached 90%. Cell experiments show CPT-CS@GO. The inhibitory effect of Fe_3_O_4_ on cancer cells was significantly greater than the use of a single drug.	[77]
CAD-GO	cephradine (CPD)	CAD-GO loaded with CPD exhibited in vitro release rates of 89.44% in PBS and 83.74% in SIF after 4 h.	[78]
GO-f-BC	Curcumin	The maximum release of curcumin reached 69.32% and can promote cell proliferation.	[79]
GO-(NH)x-SNO	NO	By rapidly releasing NO through electrical stimulation, a short-term (4 h) study on bacterial adhesion observed a reduction of over 92% in adherent bacteria, while a long-term (24 h) biofilm study found a 60% reduction in biofilm quality.	[80]
GO-HA-Ce6-GNRs	doxorubicin hydrochloride(DOX)	By combining chemotherapy, photothermal therapy, and photodynamic therapy to exert anti-tumor effects, the drug release rate reached up to 68.89%, and, compared to simple drug administration, the tumor inhibition rate was increased by 29.69%.	[81]
GOQD-PEG	Metformin	Nanoconjugates showed a sustained drug release of 72.76% (pH 5.4) and 55.9% (pH 7.4) after 24 h of study, and the nanocomposite material was able to achieve the same level of effectiveness as the free drug in enhancing glucose uptake even with a two-fold reduction in drug dosage.	[82]

## Data Availability

No new data were created or analyzed in this study.
